# Flower development: from morphodynamics to morphomechanics

**DOI:** 10.1098/rstb.2015.0545

**Published:** 2017-03-27

**Authors:** Ursula Abad, Massimiliano Sassi, Jan Traas

**Affiliations:** Laboratory for Plant Reproduction and Development, Univ Lyon, ENS de Lyon, UCB Lyon 1, CNRS, INRA, 69342 Lyon, France

**Keywords:** flower meristem, morphogenesis, molecular regulation, cell wall, modelling

## Abstract

The shoot apical meristem (SAM) is a small population of stem cells that continuously generates organs and tissues. We will discuss here flower formation at the SAM, which involves a complex network of regulatory genes and signalling molecules. A major downstream target of this network is the extracellular matrix or cell wall, which is a local determinant for both growth rates and growth directions. We will discuss here a number of recent studies aimed at analysing the link between cell wall structure and molecular regulation. This has involved multidisciplinary approaches including quantitative imaging, molecular genetics, computational biology and biophysics. A scenario emerges where molecular networks impact on both cell wall anisotropy and synthesis, thus causing the rapid outgrowth of organs at specific locations. More specifically, this involves two interdependent processes: the activation of wall remodelling enzymes and changes in microtubule dynamics.

This article is part of the themed issue ‘Systems morphodynamics: understanding the development of tissue hardware’.

## 1. Introduction

Plant development can be distinguished from animal development by a number of specific features. First of all, as plants are sessile, they have to adapt continuously their shape and architecture to their environment. As a result, they have evolved an extremely flexible mode of development. During embryogenesis, only a very rudimentary plant is formed, often composed of just a small embryonic root, an embryonic stem (hypocotyl) and a few leaves. All the rest of the plant will be elaborated from small groups of undifferentiated stem cells, called meristems, which continuously initiate new tissues and/or new organs. In this review, we will discuss the shoot apical meristem (SAM) which generates all the aerial parts of the plant. Hereby we will mainly focus on the reproductive phase, during which an inflorescence forms and the meristem generates the flowers [1]. Taking flower initiation as a case study, we will discuss recent advances in our understanding of plant morphogenesis, hereby underlining the importance of biophysical and computational approaches.

From a more cellular perspective, plant growth and morphogenesis depend on two essential processes: the control of turgor pressure and cell wall synthesis [2]. Every cell is under a high internal pressure (typically an estimated 5–10 bar in growing meristematic cells, 15–20 bar in some differentiated cells, i.e. up to 10 times that in a car tyre [3]). The cells are prevented from bursting by the presence of a robust extracellular matrix or cell wall, which counteracts the pressure inside [2]. As we will see, in order to grow the plant has to modify the cell wall structure and synthesis.

As all living beings, plants are complex systems where molecules assemble into cells, and cells into tissues and organs. These systems show emergent properties which cannot be predicted by simply considering the individual components that make up cells and tissues (for a discussion on this issue see [4]). Indeed, emergent properties also arise from the interactions between these components and the multiple feedbacks between the different levels of organization. Therefore, these systems can only be understood by analysing them at multiple scales, leading to the use of more and more interdisciplinary approaches in biology. In particular, we have seen a return of physics and mathematical modelling, but also computational biology.

## 2. A case study: the making of a flower

In many species growth is indeterminate, and many flowers can be produced by the SAM. In these species, flowers are usually initiated at the flanks of the meristems at precise positions, in highly ordered patterns. Flower primordia start as new meristems, which in turn will form the successive floral organs, often arranged in concentric whorls [5]. As most of the work in this field has been done in the model *Arabidopsis* we will mainly focus on this species. Although *Arabidopsis* has specific features, we are convinced that many of the basic principles discussed below will also apply for many other plant species.

### (a) A short overview of the molecular network that controls flower shape

#### (i) Molecular regulation of flower initiation: a central role for auxin

Extensive genetic screens combined with cellular and molecular approaches have identified a series of regulators involved in flower initiation and outgrowth (reviewed in e.g. [6]). Central for the first stages of flower formation is the plant hormone auxin. There is strong evidence coming from work on several species that auxin is concentrated at specific locations at the meristem periphery, where it induces organ initiation [7]. The accumulation of auxin is possible through the activity of membrane transporters, which often show a polar localization. As neighbouring cells frequently show a coherent distribution of these proteins, they can generate fluxes of auxin through the tissues, causing the formation of auxin maxima at certain places and auxin depletion at others [7–10]. The precise mechanisms by which the coordination of auxin transport between cells occurs is not known, but two general theoretical models have been proposed. The first model is based on the hypothesis that the cells are able to sense the concentration of the hormone and direct its flux accordingly. In this case, the behaviour of the transporters can potentially be explained by supposing that cells transport auxin against a gradient, i.e. to neighbouring cells, which have a higher auxin concentration. Interestingly, computational models in the form of virtual tissues based on this very general rule can reproduce the patterned formation of auxin maxima also observed *in vivo* [11,12]. This shows that such a mechanism is plausible, but does not exclude other hypotheses. The other model, the canalization hypothesis, proposes that cells sense and attempt to stabilize existing hormone fluxes through their membranes. The idea here is that, through a yet to be identified mechanism, active transport of auxin is increased through membranes where the net flux of auxin is positive. This feedback mechanism is able to amplify small fluxes—for example generated by diffusion—into a stable hormone flow. As for the gradient-based hypothesis, a computational model following a modified version of the canalization hypothesis also reproduces *in vivo* patterns of organ initiation [13]. So far no in-depth analysis has been performed to discriminate between these two models. This is partially because they do not propose precise cellular mechanisms, as auxin transport against a gradient or with the flux remain relatively abstract notions. Several attempts have been made to include more precise cellular processes. Thus, a model that takes into account the mechanical interactions between adjacent cells could, in principle, behave more like an up-the-gradient model. Conversely, a model where auxin movement between adjacent cells is regulated by a hypothetical receptor for auxin concentrations has points in common with the canalization model [14,15]. We must now look much more precisely into the predictions made by the different models. This will require an extensive set of quantitative experiments, in particular looking at local auxin concentrations and quantifying the distribution of auxin transporters. In addition a better understanding of the cellular processes that lead to the polarized localization of auxin transporters seems absolutely required.

#### (ii) Downstream of auxin: a complex network controlling growth patterns

**Figure 1.**
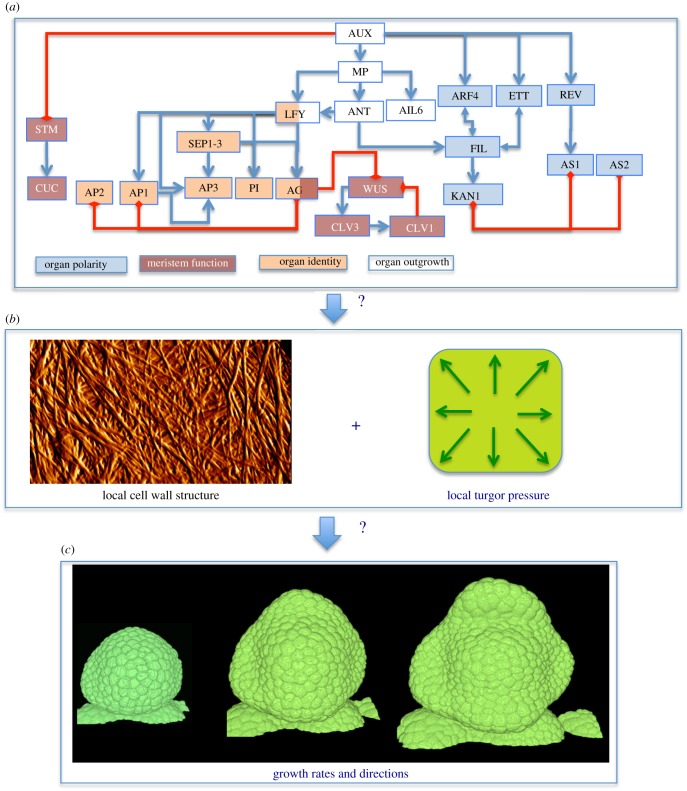
General concept for floral morphogenesis. A network of molecular, transcriptional regulators have been identified (*a*), associated with different functions (cell or organ identity, outgrowth, …). To influence growth (*b*), they must interfere with the cell wall, a complex network of cellulose fibres connected to each other via polysaccharide chains. This extracellular matrix is put under tension because of the high turgor pressure. Modifications in the cell wall make it yield to the pressure at different rates and directions. This generates the changes in shape (*c*). Graph in (*a*) based on [6] and references therein, non-exhaustive interaction graph between main floral regulators. Image in (*b*): Detail showing cellulose microfibrils in a meristematic cell wall using atomic force microscopy (G. Cloarec and J. Traas 2015, unpublished). Image in (*c*): live imaging of a growing flower bud in *Arabidopsis*. Y. Refahi and J. Traas 2016, unpublished.

Auxin feeds into a complex network of molecular regulators, which has been quite well characterized (figure 1). The core machinery of auxin-dependent gene regulation comprises the DNA-binding auxin response factors (ARFs) and their binding partners the Aux/IAAs, which recruit TOPLESS transcriptional co-repressors to ARF-bound promoters. The Aux/IAAs contribute directly to auxin perception, along with a small family of F-box proteins which target them for ubiquitination and degradation (reviewed in [16]). At the inflorescence meristem of *Arabidopsis*, the sites of incipient primordia are coincident with localized auxin accumulation and with the expression of the *AUXIN RESPONSE FACTOR5/MONOPTEROS* (*ARF5/MP*), among others [17]. It has been shown that auxin signalling guides primordium initiation via MP [18,19]. Downstream of MP, the transcriptional regulation of a group of targets has been described [20,21]. These include LEAFY (LFY) transcription factor, which is necessary and sufficient for specification of floral identity [22,23] and two members of the AINTEGUMENTA-LIKE family of APETALA 2/ETHYLENE RESPONSE FACTOR, AINTEGUMENTA (ANT) and AINTEGUMENTA-LIKE 6/PLETHORA 3 (AIL6/PLT3), which have critical roles in proliferative growth of the flower [24,25]. More recently this regulatory network of flower primordium initiation has been extended to include two more targets of MP, FILAMENTOUS FLOWER (FIL) and TARGET OF MONOPTEROS 3 (TMO3). A scenario has been proposed where transcriptional regulation downstream of auxin depends on the auxin-induced degradation of Aux/IAAs. This leads to the release of MP to regulate its targets and modifications in the chromatin state around these loci [21]. The initiation of primordia requires the downregulation of meristematic identity genes such as *SHOOTMERISTEMLESS* (*STM*) [26] as well as the demarcation of boundaries distinguished by the expression also of *STM* [27] and of the *CUPSHAPED COTYLEDON* (*CUC*) family of transcription factors [28]. Regulation of the expression of *STM* occurs at least partially via *CUC* genes and downstream of auxin [29]. The adaxial/abaxial polarity of the primordium depends on the expression of HD-ZIP III genes *PHABULOSA* (*PHB*), *PHAVOLUTA* and *REVOLUTA* (*REV*) as well as *KANADI* genes and *YABBI* (*YAB*) genes such as *FIL* [30,31].

The identity of the primordium relies on the activity of *LFY*, which positively regulates the expression of MADS-box genes proper of flower meristem identity, such as *APETALA1* (*AP1*), *CAULIFLOWER* (*CAL*) and *AGAMOUS* (*AG*). The transcriptional regulation of these and other genes leads to the establishment and development of the four whorls of the flower [32].

Notably, the gene network of primordium initiation feeds back on auxin synthesis and transport. LFY regulates auxin distribution via PINOID (PID) [20] and *AIL/PLT* genes seem to have a role in the control of transport and synthesis of auxin [33,34].

Attempts have been made to translate available experimental data about the interacting genes involved in flower morphogenesis at the inflorescence meristem into mathematical models. Diverse levels of complexity on the modelling have been undertaken. For instance, the gene regulatory network involved in *Arabidopsis* flower development [35,36] has been modelled using Boolean logic. In this case, the activity of the molecular components is assumed to be either on or off and their interactions are integrated into logical rules that describe their regulation. This approach served to produce a computational model that recovers the cellular gene expression patterns of the floral meristem that will generate sepals, petals, stamens and carpels.

More recently, the molecular regulatory network (MRN) of sepal formation was analysed using Boolean models [6]. The molecular players involved in early flower development were mapped to an atlas with cellular resolution to establish the different cell types and gene expression patterns characteristic of the floral stages at which sepal primordia emerge. Subsequently, known and supposed direct chemical interactions were used to model the network as it functions in the different cell types. Interestingly, this model was also able to account for published *indirect* interactions between elements of the MRN.

Finally, a more quantitative modelling approach of the auxin perception network at the SAM was undertaken by Vernoux *et al.* [17]. Using a set of ordinary differential equations the authors showed that robust patterning at the SAM depends not only on auxin distribution but also on the local properties of the Aux/IAA-ARF signalling network. To build this model, they used experimental evidence regarding the distribution of Aux/IAAs, ARFs and the auxin signalling machinery, as well as protein interactions identified by high-throughput yeast two-hybrid experiments [17].

### (b) How to connect this network to morphogenesis?

#### (i) The control of morphodynamics at multiple scales: linking molecular regulation to geometry

As we have seen above (§2a(ii)), auxin accumulation triggers organ initiation at least in part by feeding into a MRN. How do changes in the activity of this network lead to changes in shape at higher levels of organization? In other words, how is molecular regulation linked to geometry? This is not a trivial question and many studies do not go further than stating that a particular gene is involved in promoting or reducing growth of a particular organ. As a first essential step to address this problem, Coen *et al.* [37] proposed a straightforward concept to express gene function in terms of geometry. Starting from the fact that any change in geometry can be described by changes in local growth rates and directions, they concluded that genes locally act on three main parameters: growth rate, the degree of anisotropy and the direction of anisotropy [37]. Fundamental to their approach is that studying growth patterns as outputs of gene action requires detailed *quantitative* knowledge of geometry over time. In addition, as it is not possible to attribute precise parameter values on an intuitive basis, they designed a modelling framework for tissue growth, termed the growing polarized tissue framework [38]. The model has the form of a virtual tissue and can capture overall growth rates and directions of tissues in three dimensions, taking into account mechanical interactions between different regions. By testing different scenarios, optimal parameter values for growth rates and anisotropy can be determined that reproduce closely the geometrical changes observed *in vivo*, both in wild-type and mutant plants [39]. With this method, the authors were able to propose hypotheses for the transcriptional regulation of organ formation in different species. This framework was the first system able to simulate 3D organ development and shapes based on hypotheses for genetic regulation.

#### (ii) From morphodynamics to morphomechanics: a central role for the cell wall

Obviously, transcriptional regulators do not directly control growth rates and growth directions. For a more mechanistic analysis, we have to consider a number of specific features of plant cells, in particular those related to their physical properties. As indicated in §1, plant cells are under high internal pressure and prevented from bursting by the surrounding cell wall. This rigid wall is composed of a complex network of polysaccharides and proteins. It can be seen as a fibre-reinforced gel composed of rigid cellulose microfibrils embedded and cross-linked into a viscous matrix composed of a pectin matrix and hemicellulose chains [2,40]. Turgor pressure puts this gel under tension and growth occurs when the forces exceed a certain threshold causing the matrix elements to break and the wall to expand (plastic deformation) [41]. This is accompanied by a constant synthesis and insertion of new wall material [42]. These processes are regulated by multiple enzymes, which are in turn under control of developmental pathways [43]. The rigid cellulose fibres have mainly an inhibitory role: the more cellulose fibres per cell wall unit, the slower the cells will grow. Importantly, the cellulose fibres can be deposited in specific orientations. In that case the cells will tend to grow perpendicular to the microfibril direction. Therefore, microfibril anisotropy is a major determinant of growth anisotropy. The orientation of newly deposited cellulose fibres is under control of the cytoskeleton, in particular the microtubules, which guide the cellulose synthesizing complexes in the plasma membrane [42,44,45].

From the above it should be clear that the mechano-chemical state of the wall appears as a fundamental link between molecular growth regulation and the effective shape evolution of the tissue. In simple terms, the genetic network controlling morphogenesis can target two pathways: it can act on the enzymes that modulate synthesis and stiffness of the cell wall, or it can act on the dynamics of the cytoskeleton. A next step is, therefore, to unravel the link between developmental regulators and the structural elements of the cell. In the following paragraphs we will discuss recent progress in this field.

#### (iii) Molecular regulation of morphomechanics at the shoot apical meristem

How does auxin affect wall structure? Does it act only indirectly on the wall via transcriptional regulation? Can it also act more directly? Although we are only at the beginning of a profound understanding, several studies have addressed this issue and provide a first view of the processes at work.

*Control of wall anisotropy.* Several studies have focused on the regulation and role of wall anisotropy during organ initiation at the meristem [46–48]. As the microfibrils themselves cannot be visualized directly *in vivo*, these studies looked at microtubule dynamics which are guiding cellulose deposition [42,44,49,50]. An important feature of the growing meristem is that the outer (surface) wall seems to be loadbearing [51–54]. This hypothesis is supported by the fact that this wall is much thicker than the inner walls (typically 200–250 nm versus 80–120 nm in the *Arabidopsis* inflorescence meristem according to our own unpublished results). This importance of the outer walls in growth control of the entire meristem has an important advantage for experimentation, as the outer cells are easy to visualize *in vivo* using standard confocal microscopy.

**Figure 2.**
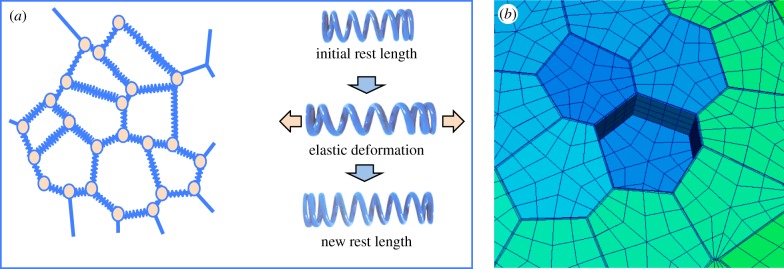
Principles used to model mechanics in virtual tissues. Many studies use spring-based models (*a*). To model plant tissues, the walls are represented as linear, elastic elements, put under tension (virtual turgor). This tension causes the springs to extend elastically. As soon as a threshold is reached, extra spring is synthesized (change in rest length), thus simulating growth. The spring-based model has its limitations, as the springs are linear, making it challenging to include mechanical anisotropy (cells being stiffer in one direction). Therefore, finite-element models seem to be more adapted. These are surfacic elements (mesh elements making up the cell walls in (*b*)), which can have anisotropic elastic properties, in contrast to the springs. Note that finite-element models require more complex software. Image in (*b*): P. Krupinski 2008, unpublished.

**Figure 3.**
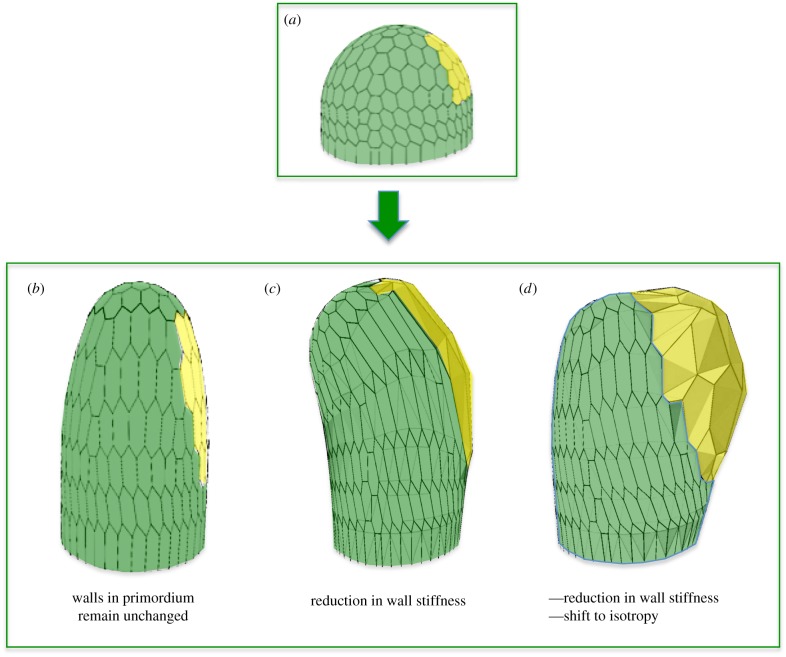
Finite-element model of a meristematic dome used to test different scenarios for organ initiation. The initial model (*a*) is a simple dome where a limited number of cells are defined as ‘primordium cells’ (in yellow). These can have different properties from the rest of the dome (green cells). Three scenarios are compared. In (*b*) all cells are anisotropic (i.e. including the primordium cells) and stiffer in the circumferential direction. When turgor pressure is increased, the dome will grow as a pin like structure. In (*c*) the cells in the primordium have a reduced stiffness (reduction of 50%). No clear outgrowth is formed and the entire structure starts to bend. In (*d*) the same reduction in stiffness is combined with a shift to complete isotropy of the walls. This causes a clear bump to grow out. The results show that a limited reduction in stiffness is synergistic with a shift to isotropy in causing outgrowths. Images based on [47]; see also for discussion [48,55].

Careful analysis of microtubule dynamics at the meristem, have revealed stereotypic behaviour and supracellular arrangements of the arrays [46,48,55]. At the tip of the SAM and of outgrowing organs, microtubules are highly dynamic, showing isotropic arrangements. Towards the flanks of the meristematic dome and along the stem, microtubules show more anisotropic arrangements, perpendicular to the apical–basal axis, in particular along organ boundaries [47,48,55]. How microtubule orientation is coordinated at the supra-cellular level is not understood. However, as the microtubules seem to align along the predicted force fields at the meristem surface, it was proposed that cells are somehow able to sense these forces and to reorganize their cytoskeleton and reinforce their walls accordingly [46,48]. Would such a mechanism be sufficient to generate the morphodynamics seen *in vivo*? To address this and other questions, several authors have turned to mechanical models in the form of virtual tissues [41] (for an overview see figure 2). Mechanical models where a feedback of forces on the cytoskeleton was simulated were able to reproduce typical morphological structures, such as a cylindrical stem or an outgrowing primordium. It is important to note that this is a typical example of self-organization: cells locally react to a mechanical signal, which more globally leads to particular shapes of the entire cell population. As long as the mechanical feedback mechanism is active, cylindrical stems will be formed (see figure 3). So how do lateral organs form? It was recently shown that during auxin-induced organ initiation at the SAM, microtubules lose their anisotropic arrangements [47]. This suggests a scenario where the outgrowth of lateral organs involves an anisotropy-to-isotropy shift. Interestingly, outgrowths form spontaneously at the surface of the meristem when microtubule arrangements are directly perturbed. This can be achieved using local applications of the microtubule-depolymerizing drug oryzalin or using mutations that perturb cytoskeleton assembly. Mutations in the microtubule severing protein katanin (in the so-called *botero1* mutant [56], which is required for the organization of microtubules in anisotropic arrays, promote outgrowth formation even in the absence of auxin transport [47]. This would suggest that a shift to isotropy might on its own be sufficient to induce lateral outgrowths. In other words, to initiate lateral organs at the SAM the cells might only have to switch off their anisotropy locally. By any means, a scenario emerges where auxin at high concentrations inactivates the capacity of the cortical cytoskeleton to organize itself along mechanical signals. As a result, these cells will start to grow out as isotropic bulges at the meristem surface. Whether this response to auxin involves transcriptional regulation is currently not known. However, katanin directly interacts with RIC1, a protein which in turn interacts with ROP6, a membrane localized cellular signalling protein involved in cytoskeleton organization [57]. It was shown that auxin treatments can activate ROP6 in young *Arabidopsis* plantlets within minutes. Although there is controversy around the signalling partners involved, this would suggest a more direct cellular link between auxin and cytoskeleton organization, probably independent from Aux/IAA and ARF transcriptional control [58–62].

*Control of wall synthesis rate and stiffness.* As indicated above, the MRNs act, in principle, not only on wall anisotropy, but also on wall synthesis rate and stiffness. Known wall-modifying proteins mainly target the matrix molecules in which the cellulose microfibrils are embedded. As indicated above, these can be roughly divided in three subcategories, (i) pectin modifying enzymes such as pectin methyl esterases (PMEs) or pectin methyl esterase inhibitors (PMEIs), (ii) enzymes targeting the hemicellulose chains such as xyloglucan endotransglucosylase/hydrolases (XTHs), and (iii) expansins which possibly target hydrogen bonds between hemicellulose and cellulose [2]. Little is known about the precise molecular regulation of these, but among the published potential targets of many transcription factors there are a range of wall modifiers [63–66].

Whereas many mutants perturbed in wall synthesis have been described, very few of them have been characterized at the level of the SAM. Nevertheless, experiments where the activities of some of these modifying enzymes are manipulated show their potential importance in organ formation at the shoot apex. Fleming *et al.* [67] performed local applications of expansins on SAMs of tomato, thus inducing the formation of ectopic leaf-like organs. More recently, Peaucelle *et al*. [65,68,69] have explored the possible roles of PMEs and PMEIs on organ formation at the inflorescence meristem of *Arabidopsis*. These enzymes control the stiffness of the pectin matrix. Antibodies recognizing specific modifications in pectin chains indicated that specific meristematic zones, in particular organ boundaries, are likely to have a stiffer pectin matrix. Interestingly, overproduction of PMEI completely inhibited organ formation at the shoot apex, whereas local applications of PME induced extra flowers.

Both the experiments using expansins and PME/PMEIs point to a scenario where matrix molecules are stiffened or loosened at sites where growth has to be respectively inhibited as in organ boundaries or increased as in organ primordia. Several attempts have been made to test this hypothesis using atomic force microscopy (AFM) to measure the local stiffness of the walls. Depending on the thickness of the AFM probes used, different results were obtained. Thicker probes (1–5 µm) indicated that the inner walls became more elastic at the moment of organ initiation. However, when very small probes (10 nm) were used to measure only the supposedly limiting outer wall, relatively minor differences were observed, not exceeding a reduction of 20–50% in stiffness in the very young initium [47,65].

#### (iv) Integrating the current data using mechanical models

The results described above (§2b(iii)) indicate that both changes in wall anisotropy and changes in wall stiffness are associated with organ initiation at the meristem. How plausible is this? Are the observed changes in stiffness sufficient? Why two types of modification? To address these questions mechanical models were again extremely useful [47]. Finite-element models (figures 2 and 3) of a simplified meristematic dome were used to explore the potential effects of the observed changes in wall anisotropy and stiffness on lateral outgrowth (figure 3). In a first series of simulations, the walls at the meristem surface were highly anisotropic, being much stiffer in the circumferential direction. If this structure is simulated to grow, it forms a cylindrical structure, as expected. A next simulation where a small number of cells at the flank of this dome were instructed to reduce the stiffness of their periclinal walls by 50% was unable to produce a clear lateral outgrowth. Interestingly, when such a modest reduction in stiffness was *combined* with a local shift to cell isotropy a frank lateral outgrowth was produced. This suggests that growth isotropy has the capacity to amplify the effect of minor reductions in cell wall stiffness to increase cell growth rates in the bulging primordium [47].

## 3. Concluding remarks and perspectives

**Figure 4.**
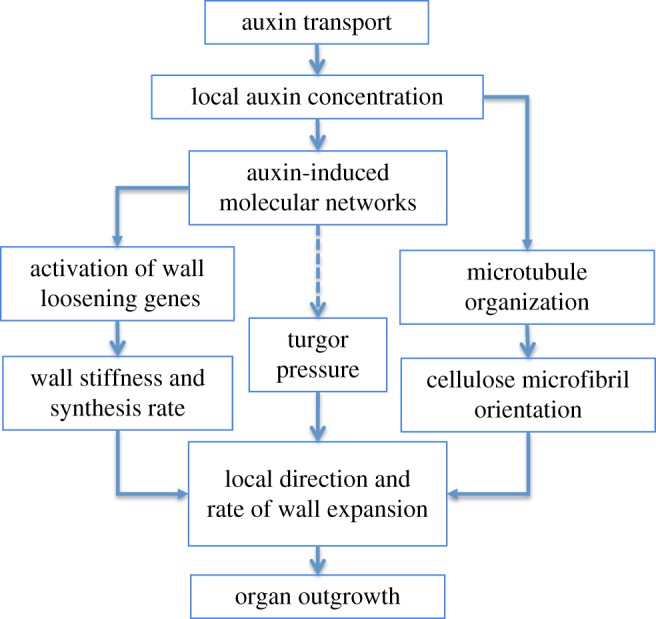
Scenario proposed for organ initiation. Auxin transport causes auxin accumulation. This has a dual effect. First microtubules disorganize, causing a shift to isotropic cellulose deposition. Second, wall stiffness is slightly reduced. Together, these two effects lead to increased growth rates and organ outgrowth. Growth is driven by turgor pressure but how this is regulated is not known (dashed arrow). Feedbacks (e.g. between molecular networks and auxin concentration) or mechanical feedbacks on the cytoskeleton are not represented.

Our current knowledge leads to a scenario where both physical and biochemical properties generate the shape changes observed during auxin-induced organ formation at the SAM (figure 4). Without auxin transport at its surface, the growing meristem is in a default state, only producing a naked stem. The existing evidence suggests that this is due to a mechanical feedback, where every cell resists the dominating direction of the force pattern. How this precisely works is not known, but somehow the cell must sense this stress and orient its microtubules accordingly [46,70]. In turn the cytoskeleton will then reinforce the cell wall along the force vector. To induce an organ, auxin must first accumulate locally. This accumulation temporarily switches off the feedback causing the cells to bulge out isotropically. In parallel, the effects of growth isotropy are reinforced by a relatively modest reduction in wall stiffness. It is noteworthy that the establishment of plant architecture thus would emerge from two local cellular properties: (i) the capacity of the cells to resist forces, and (ii) the ability to orient the auxin fluxes along local polarized information (auxin gradients or directional fluxes). Whereas this general scenario would explain the basic, modular development of plants in branched structures, it remains to be established how the precise shape of the organs is determined once they are initiated.

In the near future a range of scientific questions and technical issues need to be addressed. Existing models propose changes in auxin concentrations, cell wall composition and structure. Unfortunately it remains very difficult to obtain detailed quantitative information on these parameters. Some progress has been made in the detection of auxin distribution and perception [71], but we are still a long way from what is ideally needed, which should include both intra- and extracellular concentrations. Likewise, the dynamics of wall properties (in particular wall composition), have remained difficult to assess at the shoot apex. From a more scientific point of view, we still know very little about the mechanisms that control auxin transport and cytoskeleton organization. In particular the link between physical constraints and cytoskeleton organization has remained elusive.

From a more theoretical point of view, the development of more sophisticated models needs to proceed. Past years have seen significant advances in the development of mechanical models at multiple scales (see also [72,73], both in this issue). The first three-dimensional models of multicellular tissues have now been developed, making it possible to compare in more detail theoretical predictions and experimental outputs. These models should now be extended, in the first place integrating simple molecular circuits.
